# Integration failure of regenerated limb tissue is associated with incongruencies in positional information in the Mexican axolotl

**DOI:** 10.3389/fcell.2023.1152510

**Published:** 2023-06-02

**Authors:** Warren A. Vieira, Michael Raymond, Kristina Kelley, Matthew A. Cherubino, Hande Sahin, Catherine D. McCusker

**Affiliations:** McCusker Lab, Biology Department, University of Massachusetts Boston, Boston, MA, United States

**Keywords:** axolotl, limb regeneration, integration, accessory limb model, bulbous mass, retinoic acid, limb patterning

## Abstract

**Introduction:** Little is known about how the newly regenerated limb tissues in the Mexican axolotl seamlessly integrate with the remaining stump tissues to form a functional structure, and why this doesn't occur in some regenerative scenarios. In this study, we evaluate the phenomenological and transcriptional characteristics associated with integration failure in ectopic limb structures generated by treating anterior-located ectopic blastemas with Retinoic Acid (RA) and focusing on the “bulbus mass” tissue that forms between the ectopic limb and the host site. We additionally test the hypothesis that the posterior portion of the limb base contains anterior positional identities.

**Methods:** The positional identity of the bulbus mass was evaluated by assaying regenerative competency, the ability to induce new pattern in the Accessory Limb Model (ALM) assay, and by using qRTPCR to quantify the relative expression of patterning genes as the bulbus mass deintegrates from the host site. We additionally use the ALM and qRTPCR to analyze the distribution of anterior and posterior positional identities along the proximal/distal limb axis of uninjured and regenerating limbs.

**Results:** The bulbus mass regenerates limb structures with decreased complexity when amputated and is able to induce complex ectopic limb structure only when grafted into posterior-located ALMs. Expressional analysis shows significant differences in *FGF8*, *BMP2*, *TBX5*, *Chrdl1*, *HoxA9*, and *HoxA11* expression between the bulbus mass and the host site when deintegration is occuring. Grafts of posterior skin from the distal limb regions into posterior ALMs at the base of the limb induce ectopic limb structures. Proximally-located blastemas express significantly less *HoxA13* and *Ptch1*, and significantly more *Alx4* and *Grem1* than distally located blastemas.

**Discussion:** These findings show that the bulbus mass has an anterior-limb identity and that the expression of limb patterning genes is mismatched between the bulbus mass and the host limb. Our findings additionally show that anterior positional information is more abundant at the limb base, and that anterior patterning genes are more abundantly expressed in proximally located blastemas compared to blastemas in the more distal regions of the limb. These experiments provide valuable insight into the underlying causes of integration failure and further map the distribution of positional identities in the mature limb.

## 1 Introduction

As regenerative biologists edge closer to stimulating an endogenous regenerative response in damaged human limbs, there are still many aspects regarding the basic biology of limb regeneration that are unknown, yet essential, for the generation of fully functional human limb regenerates. One crucial aspect of limb regeneration requiring further study is the integration of the newly formed tissues with the existing limb tissues. We define integration as the process by which the pattern of the newly regenerated (or grafted) tissues align seamlessly with the existing pattern in the surrounding tissues (Vieira and McCusker, 2018). Although there are many tetrapod species capable of regenerating fully functional limbs, very little is known about how the various types of limb tissues integrate following a regenerative response. Additionally, we are just beginning to understand how some regenerated structures fail to integrate properly. Thus, these species can provide valuable insight into the biology underlying this important process.

In amphibian models, the initiation of limb regeneration occurs when axons innervate a layer of wound epithelium covering the wounded limb tissue (Singer, 1978). This results in the formation of a nerve-dependent, specialized signaling center within the distal wound epithelium that leads to the dedifferentiation of the underlying mature limb tissues, the recruitment of these cells to the site of injury, and the reactivation of the cell cycle to form the transient regenerative organ known as the blastema (McCusker et al., 2015; Duerr et al., 2022). For the new/missing pattern to emerge in the regenerating tissue, connective-tissue derived cells from both anterior and posterior limb locations need to interact in the blastema. While multiple models (intercalary or gradient-based) have been proposed to explain how this occurs, the underlying molecular mechanism driving new pattern formation in the regenerate is not fully understood, but likely includes multiple steps (Bryant et al., 1981; Meinhardt, 1983; Vieira and McCusker, 2019). Regardless, as the blastema tissues pattern and redifferentiate into the missing structures, they simultaneously integrate with the limb tissue that they are growing on.

The integration of the regenerated tissues in a complex structure, such as a limb, requires more than the fusion between the newly formed and existing tissues as what occurs with the skeletal and muscle tissue (Nacu et al., 2013; Vieira et al., 2018; Riquelme-Guzmán et al., 2022). In these tissues, the degeneration of the mature tissues close to the developing blastema likely plays an important role in integrating the new with the old (Sandoval-Guzmán et al., 2014; Riquelme-Guzmán et al., 2022). Moreover, complete integration also requires the reconnection of long-distance tissue interactions that may span over the site of injury, such as those that occur with ligaments, tendons, and the peripheral nervous system (Song et al., 2019). Last, these structures need to integrate seamlessly into restored limbs, regardless of where they were amputated along the proximal/distal axis. This means that the mechanism of tissue integration is somehow coupled with the positional information within the zone of integration (reviewed in (McCusker and Gardiner, 2014; Vieira and McCusker, 2017).

**FIGURE 1:**
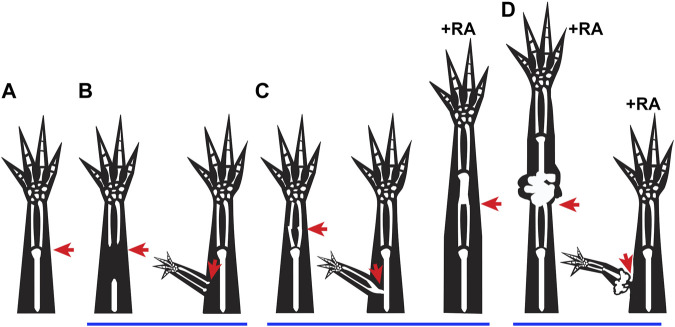
Integration failure phenotypes: **(A–D)** Cartoons represent previously observed integration phenotypes in regenerating salamanders, where the red arrows indicate the site of injury. **(A)** Representation of normal integration of the regenerated tissue (distal to injury site) with the stump tissue (proximal to injury site) following a limb amputation. **(B)** Incomplete joint formation between the regenerated and existing tissue when proximal skeletal elements are removed from the stump prior to amputation (Weiss, 1925), and when an ectopic limb is formed following an ALM surgery (Endo et al., 2004). **(C)** Fused integration phenotypes are observed when bone processing or maturation are altered (Vieira et al., 2018; Riquelme-Guzmán et al., 2022), when a deep injury is made during ALM Surgery (Makanae et al., 2014a), and occasionally when an amputation blastema is treated with RA (Thoms and Stocum, 1984). **(D)** Disruption of integration due to growth of ectopic tissue is observed when amputation blastemas are treated with high doses of RA (Thoms and Stocum, 1984), or when an ectopic anterior blastema is treated with RA (McCusker et al., 2014).

While under normal circumstances, tissue integration occurs simultaneously with regeneration, there are a number of examples throughout the literature where the integration of the regenerate is defective, showing that these two processes are not necessarily coupled (Figure 1) (McCusker and Gardiner, 2014; Vieira and McCusker, 2017). The failure to align the pattern of the skeletal tissues has most widely been reported on and, based on the final structures that form, can roughly be categorized into three integration failure types: incomplete or hemi joints (Figure 1B) (Weiss, 1925; Goss, 1956; Makanae et al., 2014a), skeletal fusions (Figure 1C) (Satoh et al., 2016; Vieira et al., 2018; Riquelme-Guzmán et al., 2022), and disruption by amorphic cartilage mass (Figure 1D) (Thoms and Stocum, 1984; Crawford and Stocum, 1988a; Ju and Kim, 1994; McCusker et al., 2014; Vieira et al., 2019). With the exception of some skeletal fusion phenotypes that are linked to altered processing of the bone in the stump tissue or differentiation of the new skeletal tissue (Vieira et al., 2018; Riquelme-Guzmán et al., 2022), most of the reported skeletal integration-failure phenotypes can be traced back to treatments that lead to aberrant positional information between the new and old tissues. Misaligned positional information has also been linked to the integration failure of engrafted skeletal tissue in mammals (Leucht et al., 2008). This indicates that studying the underlying causes of integration failure of amphibian regenerates could lead to the development of more successful regenerative strategies for humans.

Multiple examples of skeletal integration failure are caused by treating blastemas with exogenous retinoic acid (RA), a morphogen that can regulate the expression of a number of patterning genes in the regenerating limb field (Bryant and Gardiner, 1992; Scadding and Maden, 1994; Gardiner et al., 1995; Mercader et al., 2005; Wang et al., 2009; Lee and Gardiner, 2012; Monaghan and Maden, 2012). (Also include James and Tim’s recent agonist paper). RA treatment leads to the re-specification of proximal/distal (P/D) and anterior/posterior (A/P) positional information in the undifferentiated blastema tissue (Thoms and Stocum, 1984; Kim and Stocum, 1986a; Bryant and Gardiner, 1992; Vieira et al., 2019). When an amputation blastema is treated with RA, the positional information is reprogrammed to a more proximal positional identity, and leads to the duplication of the proximal limb segments that either fuse with the existing skeletal tissue, or are physically separated by an amorphic mass of cartilage called the “bulbus mass” (Maden, 1983; Thoms and Stocum, 1984; Crawford and Stocum, 1988a).

**FIGURE 2:**
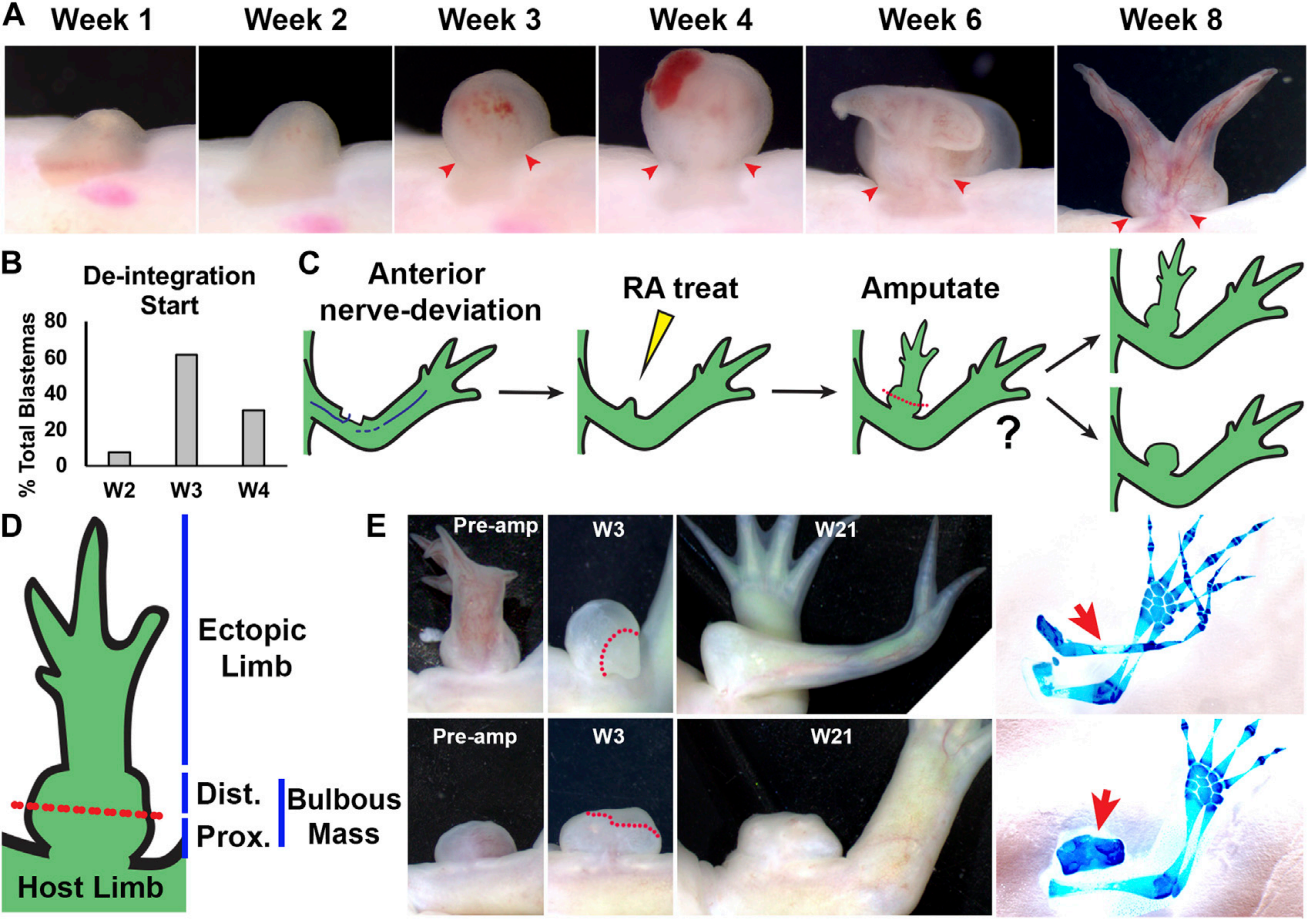
Characterization of RA-induced ectopic tissue in innervated anterior wound sites: **(A)** Live image time course over 8 weeks of anterior-located blastemas treated with RA showing bulbous mass formation, and de-integration from the host limb. Red arrows mark the location where the bulbous mass is connected to the host limb at the time points when the bulbous mass is wider than the site of connection. **(B)** Histogram showing the time point when the RA treated blastemas (shown as % of the total blastemas) exhibited de-integration (N = 13) **(C)** Cartoon describing experiment design. Anterior located innervated limb wounds will generate blastemas, which upon systemic treatment with RA, will form ectopic structures connected to the host via bulbous mass tissue. Ectopic structures were amputated through the bulbous mass and were monitored for regeneration. **(D)** Diagram of RA-induced ectopic limb structure and bulbous mass. Red dotted line indicates the position of the amputation plane in the regeneration assay in **(E)**. **(E)** Live images of RA-induced ectopic limb/bulbus mass (top) and RA-induced bulbous mass alone (lower) before amputation (Pre-amp), and 3 and 21 weeks (W3, W21) post-amputation. Dotted red line delineates the boundary between the remaining bulbous mass and the blastema. (right) The resulting ectopic structures are indicated with red arrows in the whole mount skeletal stained limbs. Table 2 has the full experimental statistics for **(E)**.

One of the most extreme examples of integration failure occurs in the axolotl model when an anterior-located ectopic blastema, generated by deviating the brachial nerve bundle into a superficial wound site on the forelimb, is treated with RA (McCusker et al., 2014; Vieira et al., 2019). Within 7 days of RA treatment, the ectopic blastemas have “posteriorized” gene expression patterns*,* and eventually form ectopic limb structures with complete P/D and A/P axes (Vieira et al., 2019). Although the respecified blastemas exhibit a robust regenerative response, the ectopic limb structure grows on a bulbous mass, which itself interferes with skeletal integration. Moreover, over time, the bulbus-mass tissue de-integrates from the host limb, only remaining connected with a thin bridge of tissue containing mostly skin (Figure 2) (McCusker et al., 2014). This is interesting because the ectopic limbs that are induced from a posterior skin graft, as opposed to RA treatment, in the same type of wound are well-connected at the soft tissue level (Endo et al., 2004) (Figure 1B). Thus, RA-induced ectopic limbs exhibit integration failure at both the skeletal/joint and soft tissue levels, which to our knowledge has not been previously observed in the limb. This provides a unique system to understand the contribution of positional information in the integration of both tissue types simultaneously.

Using grafting assays, regenerative assays, and expressional analyses on RA-induced ectopic limbs, we demonstrated that these structures have stabilized anterior and posterior positional information (Vieira et al., 2019). However, the positional content of the bulbus mass tissue, which is the region of the ectopic tissue that de-integrates with the host limb, is not known. One possibility is that RA reprograms the cells that generate the bulbus mass to a positional identity proximal/outside of the limb field. The observation of structures that appear similar in shape to the shoulder girdle have been previously reported on the proximal end of the limbs structures that are generated from RA treated amputation blastemas (Thoms and Stocum, 1984; Kim and Stocum, 1986b). Additionally, engraftment of shoulder girdle cartilage into blastemas on irradiated hosts results in the generation of amorphic cartilage structures, reminiscent of the bulbus mass, that fail to integrate with the host skeleton (Eggert, 1966). Another possibility that is not necessarily incompatible with the one mentioned above, is that exogenous RA has “off target” effects that effectively scramble the positional information in the treated cells. Thousands of Retinoic Acid Responsive Elements (RAREs) sequences are present throughout the vertebrate genome, and of the ones that have been validated as *bona fide* RA targets (Lalevée et al., 2011), only a small number are specifically linked to activities in the limb field (reviewed in (Cunningham and Duester, 2015). While this latter possibility could explain the bulbus mass phenomenon, it is confounding how ectopic limb structures with normal pattern and positional memories are also generated from the RA-reprogrammed tissue (McCusker et al., 2014).

To shed light on these possibilities, the current study focused on the positional context at the site of bulbus mass de-integration from an anterior forelimb stylopod host site. We evaluated the A/P positional information in the bulbus mass by testing the regenerative and pattern inducing abilities of the tissue and found that it predominantly behaves like anterior limb tissue. Using qRT-PCR to evaluate expressional differences in genes differentially expressed along the P/D limb axis or between the limb field and flank tissue, we identified significant differences between the bulbus mass and stylopod. Importantly, the bulbus mass robustly expresses genes associated with limb identity (*Tbx5, HoxA9, HoxA11),* indicating that it is composed of limb tissue. However, from the current analysis we were unable to determine with confidence whether the bulbus mass also contains positional information from outside of the limb field. Together these findings indicate that incongruencies of P/D positional information are present between the bulbus mass and the host site while de-integration of the soft tissue is occurring.

The above-described observation that the bulbus mass is composed of anterior limb information, in conjunction with previously published phenomenological observations from Gardiner and Bryant that show that A/P information is asymmetrically distributed in the limb skeletal segments, led us to hypothesize that anterior positional information is more abundant (at the expense of posterior information) in the most proximal regions of the limb (Gardiner and Bryant, 1989). We tested this possibility phenomenologically using the ALM surgery in different regions of the limb base and with a qRT-PCR-based expression analysis of anterior and posterior patterning genes in blastemas from different P/D limb locations. The outcomes of both experiments indicate that anterior information is more abundant in the proximal limb locations and could provide important insight on how a segment-appropriate A/P pattern is established when the limb is amputated in different limb positions.

## 2 Materials and methods

### 2.1 Animal husbandry

White (RRID: AGSC_101J) and GFP strain (RRID: AGSC_110J) Mexican axolotls, spawned on site at UMass Boston or obtained from the Ambystoma Genetic Stock Center at the University of Kentucky, were utilized for this study. All experimental work was approved by the Institutional Animal Care and Use Committee of the University of Massachusetts, Boston and conducted in accordance with the recommendations in the Guide for the Care and Use of Laboratory Animals of the National Institutes of Health. Prior to surgical manipulation and/or tissue collection, animals were anesthetized by administering 0.1% MS222 solution (Ethyl 3-aminobenzoate methanesulfonate salt, Sigma), pH 7. 0. All experimental animals were between 14 and 20 cm, snout to tail tip.

### 2.2 Surgeries

Generation of bulbous mass tissue: Bulbous mass tissue can be generated reproducibly by utilizing a modified version of the ALM established by McCusker *et al* (McCusker et al., 2014). Briefly, innervated wound sites were created by deviating the brachial nerve to 2 mm × 2 mm lateral wounds located on the anterior side of the zeugopod of the forelimb on GFP + animals. The animals were kept on ice post-deviation for 1.5 h to promote nerve retention in the site, misting every 5–10 min with housing water to prevent desiccation. Once mid-bud stage blastemas formed (approximately 7–9 days post-surgery), animals were injected intraperitoneally with 150 µg of RA per Gram of body weight. The wound sites were monitored closely for an ectopic regenerative response and only those sites that produced ectopic structures were assessed further (80% of sites generated ectopic patterns). All ectopic structures had a bulbous (amorphic) mass from which additional limb structures may have extended. The positional information of the bulbous mass was then assessed in three ways – by 1) amputation and 2) ALM grafting experiments to determine the limb-specific positional diversity of the structure; and 3) by transcriptional analysis to further characterize the positional information.

Bulbous mass amputations: To evaluate whether the bulbous mass contained enough positional diversity to regenerate limb structures, bulbous masses (21 weeks post-surgery) were amputated. Bulbous masses, six with pattern outgrowths and three constituted solely by bulbous mass tissue according to live assessment, were amputated proximally (region closest to the limb proper). The amputated structures were monitored for blastema formation and then allowed to regenerate. Regeneration was considered complete (patterned and differentiated) when fully developed skin covered the regenerates. At week 21, the tissues were collected, and pattern was assessed by cartilage-specific whole mount staining.

Bulbous mass grafts into ALM: To determine whether the bulbous mass tissue contained both anterior and posterior positional information, pieces of the mass including skin and mesenchyme (21 weeks post-surgery) were grafted into a standard ALM (Endo et al., 2004) on anterior or posterior innervated wound sites in the mid-stylopod as reported previously (Nacu et al., 2016; Vieira et al., 2021). Briefly, 2 mm × 2 mm lateral wounds were generated on the anterior or posterior of white-strain axolotl forelimbs. Innervation was achieved by deviating the brachial nerve to these sites. Animals were maintained on ice for 1.5 h post-surgery to promote nerve retention in the wound site. Two days later, once a wound epithelium had been generated, the collected GFP + bulbous mass tissue was implanted into the wound site, under the wound epithelium. The objective of this 2-day waiting period was to allow the wounds to develop a wound epithelium that could be used as a “pocket” to place the grafted tissue, which improved graft retention in wound site.

Due to the amorphous nature of the bulbous mass, the grafts were harvested by collecting full thickness skin (dermis and epidermis) from both the proximal (closest to the host limb) and distal (farthest point from the host limb) regions of the structure. Caution was taken to ensure that the collected tissue was not derived from a region of ectopic limb that may have extended from the bulbous mass itself; as previous studies show that these ectopic limb regions encompass both anterior and posterior positional information (Vieira et al., 2019). Post-grafting, the animals were maintained on ice for 1.5 h, misting regularly, to promote graft retention. The wound sites were monitored on a weekly basis for a regenerative response. Any wound site that lost the graft and/or failed to generate a blastema within 3 weeks post-surgery were excluded from further analysis. Fluorescent assessment in the live animals allowed for tracking of the grafted tissue (GFP^+^) in the wound sites. Regeneration was considered complete (patterned and differentiated) when fully developed skin covered the regenerates. At week 21, the tissues were collected, and skeletal pattern was then assessed by whole mount skeletal staining.

Limb base nerve deviations and ALMs: To perform nerve deviations at the limb base, the brachial nerve was deviated to approximately 4 mm × 4 mm square anterior or posterior stylopod wound sites overlapping the juncture between the flank and the forelimb in white animals. Only wound sites that generated a blastema within the first 3 weeks were considered for further analysis. To perform limb base ALMs, fresh nerve deviations as described above received a graft of GFP + skin, approximately 2 mm × 2 mm harvested from anterior or posterior sides of the autopod (located next to the carpals) into the anterior or posterior wound sites, respectively. Only the wound sites that generated a blastema and retained GFP + grafts through week 3 post-surgery were included for further analysis. Animals were monitored for ectopic pattern formation until week 20, when the limbs were harvested for whole mount skeletal staining.

### 2.3 Whole mount skeletal staining

Victoria blue whole mount staining, as described in Bryant and Iten (Bryant and Iten, 1974), as well as Alcian Blue and Alizarin Red whole mount staining, as described in Horton and Maden (Horton and Maden, 1995) were used to visualize cartilage patterns within the collected tissues. The ectopic cartilage formed in the ALM wound sites were scored based upon the number of skeletal elements documented. The following classifications were used, as described in Vieira et al. (2019) - no element, single nodule, multiple symmetrical elements, multiple asymmetrical elements and complete limb.

### 2.4 Gene expression analysis

Transcriptional analysis was performed on bulbous mass tissue to characterize positional information. This analysis was conducted over the course of bulbous mass development to document any temporal changes in marker expression profiles within this tissue. RA induced bulbous masses were generated as described above, and allowed to develop over 11 weeks, at which time the ectopic pattern was fully differentiated, patterned, and covered by mature skin. Tissue was collected at the following time points: week 0 (day of RA treatment, but prior to RA treatment), one, five, nine, and 11 post RA treatment. Due to tissue size, one biological replicate was constituted by four wound sites for weeks zero, one and five, two wound sites for week nine, and one wound site for week 11. For each time point a total of 4 biological replicates were collected. Full thickness mature skin was also collected from the axolotl flank, posterior and slightly ventral to the limb, and from the stylopod, zeugopod, and autopod of the forelimb. For the analysis on the amputation blastemas along the P/D limb axis, forelimbs were amputated at the three positions along the proximal-distal axis: wrist-level, mid-zeugopod level, and mid-stylopod level, and the protruding bone was trimmed. Blastemas were allowed to develop to the mid-bud stage, at which point they were harvested for qRTPCR. Four blastemas were pooled for each biological replicate, and 4 biological replicates were performed for each limb location.

The collected tissue was put in Tripure Isolation Reagent and the total RNA was purified using the Nucleospin RNA XS kit (Macherey-Nagel), according to the manufacturers’ specifications. The mRNA was then converted into cDNA libraries using the Transcription first strand cDNA synthesis kit (Roche), in conjunction with the anchored oligo (dT)18 primer, as specified by the manufacturer. Relative expression of flank and limb enriched markers, relative to that of the housekeeping gene *Ef-1α*, in each experimental sample was then assessed by qPCR (Azuraquant™ Green Fast qPCR Mix Lo-Rox, Azura Genomics Inc.), using the Pfaffl method (Pfaffl, 2001). The expressional abundances between samples were compared using a two-tailed T-test assuming equal variance. Positional markers were initially identified in literature and the primers were validated using cDNA libraries generated from axolotl flank and forelimb (stylopod) tissues (Nacu et al., 2016; Purushothaman et al., 2019, 2022; Vieira et al., 2019; Vincent et al., 2020). Primer sequences for all the transcripts analyzed are in Table 1.

**TABLE 1:** qRTPCR primer sequences.

Gene	Forward primer (5′to 3′)	Reverse primer (5′to 3′)
***Alx4***	GGA​CAC​CCA​GAA​TAC​TGA​GCA	TTA​AGT​GCC​CTG​TCA​TGT​GG
***BMP2***	ATG​CCA​TCG​TGC​AGA​CTT​TG	ACC​ACC​TTT​TCG​TTC​TCG​TC
***Chrdl1***	GAG​CCC​TAC​GGA​TTG​GTG​TA	GTG​GAT​AGG​GTT​GGC​ACA​GT
***Ef1-a***	AAC​ATC​GTG​GTC​ATC​GGC​CAT	GGA​GGT​GCC​AGT​GAT​CAT​GTT
***FGF8***	AGCTGATTGGCAAGAGCA	GTG​AAG​GCC​ATG​TAC​CAT​CC
***Gli3***	CAT​GGA​TGT​GGT​CGT​TAT​TGA​TGT​G	GAG​GTT​ATT​TAC​GAG​ACC​GAC​TGT​C
***Grem1***	GGA​CAC​CCA​GAA​TAC​TGA​GCA	GTA​GAC​CAA​TCG​AAA​CAT​CCT​GT
***Hand2***	CCA​GCT​ACA​TCG​CCT​ACC​TC	AGC​TCT​TTC​TTG​CGC​TTG​TC
***HoxA9***	CTG​GGC​CTT​TTA​TTG​TGC​AT	GTG​ACA​TCT​GAG​TGC​GTT​CG
***HoxA11***	GTG​TTG​CCA​AAC​AGG​CTT​GG	TAG​TCA​TTT​CCT​GCA​CGA​AGG​TC
***HoxA13***	CAG​GAG​GGT​CAA​AGA​GAA​GAA​GG	TCT​GGT​GAC​CAA​TGA​ACA​GCT​TC
***HoxC5***	CGG​CAG​ATC​AAA​ATC​TGG​TT	GCC​AGA​ATG​ACC​GAT​TCA​CT
***Ptch1***	TGT​AGA​TCT​GCT​CCA​ATG​CAA​AC	CTG​ACC​CGG​AGT​ACT​TGC​AG
***Shh***	AGT​GAC​CAG​ACC​GGT​GAA​AG	CGC​TCC​GTC​TCT​ATC​ACG​TA
***Tbx5***	CTG​GAA​GGC​GCA​TGT​TTC​CAA​GTT	TGG​CGA​ATC​CGG​ATG​GAC​GTA​TAA

### 2.4 Prediction of axolotl RARE binding motifs

Genomic regions of interest for target genes were queried using the UC Santa Cruz Genome Browser Gateway on *Axolotl Assembly V6, 2020* on the largest Full Gene Model annotated. The genomic sequences for the promoter and 1,000 upstream bases (Upstream Region), the 5′UTR Exon, Intron, and 3′UTR Exon were mined and run through Clustal Omega against a list of previously identified vertebrate RARE motifs (Cunningham and Duester, 2015). Any predicted alignments of 10/13 or more, 11/14 or more, and 13/17 or more for 1DR, 2DR, or 5DR motifs, respectively, were analyzed further. Identified motifs that had ≥4 exact matches in each of the DR regions of the motif were deemed to be predicted RARE motifs.

## 3 Results

### 3.1 RA-induced ectopic limb structures de-integrate from the host limb

We have previously shown that treatment of anterior-located ectopic blastemas with RA induces the formation of complete ectopic limbs that fail to integrate with the host limb site (McCusker et al., 2014; Vieira et al., 2019). Although the underlying cause of integration failure is unknown, it is no doubt in part due to the formation of a large bulbous mass that separates the new structure from the existing limb tissues of the host site. The bulbous mass itself differentiates into amorphous masses of cartilage and connective tissue surrounded by mature skin but is almost completely lacking muscle and pigment cells (McCusker et al., 2014). This is interesting because both muscle and pigment cells follow positional cues from connective tissues (Nacu et al., 2013; McCusker et al., 2014) and they likely traveled *through* the bulbous mass to contribute to the ectopic limb structures that grow on top of it. Upon more detailed observations, we found that the bulbous mass tissue gradually “de-integrates” from the host limb, such that only a thin bridge of soft tissue connects the two (McCusker et al., 2014).

We hypothesized that either irreconcilable differences in positional information or mechanical forces generated by the bulbous mass itself could lead to the diminishment of integration. Previous studies by Crawford and Stocum showed that when blastemas from different locations along the P/D limb axis are grafted to a proximally amputated host limb, the grafted blastemas continue to develop and integrate into location-appropriate positions on the host regenerate (Crawford and Stocum, 1988b). Thus, complimentary positional information within the limb field appears to facilitate the integration of the grafted blastemas with the host limb tissues. With this in mind, one possible explanation for the above-described observations is that RA treatment, which is known to reprogram the positional information in blastema cells to a proximal limb identity (Thoms and Stocum, 1984; Niazi et al., 1985; Crawford and Stocum, 1988b; Bryant and Gardiner, 1992; Maden, 2000; Nguyen et al., 2017), could reprogram the positional information to somewhere proximal of the limb field. Alternatively, RA treatment may lead to a mismatch of limb positional information between the bulbous mass and the host site.

It is also conceivable that the large size of the bulbous mass could generate mechanical forces that somehow narrow the tissue connecting it to the host site. Mechanical forces are known to play important roles in shaping tissue during embryogenesis, and long-term application of mechanical force is a well-used tool in the shaping of differentiated structures in humans, such as the use of braces to repair orthopedic deformities (Su and Nan, 2014). To provide insight into this possibility we documented the development of the bulbous mass to determine the time point in which the narrowing of the connection between the mass and the host limb begins (Figure 2A).

Animals with ectopic blastemas that were generated by deviating a nerve to an anterior-limb wound site, were injected with RA 1-week post-surgery as described previously (McCusker et al., 2014) and the girth of the connection and bulbous mass tissues were assessed visually weekly. In this context, we define “narrowing” to occur when the girth of connection is narrower than the girth of the bulbous mass. We observed narrowing of the connecting tissue as early as 2 weeks, with most bulbous masses narrowing by 3 weeks post RA injection (Figures 2A, B). After these time points, the bulbous mass grows rapidly, continues to de-integrate from the host limb, and typically between 5 and 6 weeks will develop new blastema-like bumps on its distal surface that will eventually form the ectopic limbs. Because the bulbous mass is relatively small at the time when the narrowing of its connection occurs, we reasoned that it is unlikely that mechanical forces play a major role in de-integration from the host limb, at least during these early stages of development. Therefore, we decided to further pursue the possibility that positional discontinuities contribute to this process.

### 3.2 Phenomenological characterization of positional information in the bulbous mass tissue

To test whether the bulbous mass was composed of tissue with positional information from within the limb field, we performed regeneration-based assays to evaluate the ability of the bulbous mass to generate limb structures. We have previously shown that RA-induced ectopic limbs will regenerate if amputated proximally (Vieira et al., 2019). For this to occur, the ectopic limbs need to contain cells with positional information from the different limb axes (Bryant et al., 1981; Endo et al., 2004; Nacu et al., 2016). The Satoh lab previously observed that if regeneration permissive signals are moved to just outside of the limb field, ectopic limbs will not form unless limb tissue is grafted into the site (Hirata et al., 2013). We reasoned that if the bulbous mass has an identity outside of the limb field, then it will not regenerate limb structures, even if the conditions support blastema formation. Bulbous masses were generated as previously described, and at week 21, were amputated midway through the bulbous mass and allowed to regenerate (Figures 2C–E). Six of the bulbous masses had ectopic limb structures, and three did not (Table 2). All amputations led to the formation of blastema-like bumps (Figure 2E, Table 2), but only the bulbous masses that were connected to an ectopic limb were able to regenerate limb structures (Figure 2E; Table 2). We note, however, the limb regenerates that formed were less complex in A/P structure than the original ectopic limb structures (Figure 2E). While it was difficult to fully assess regeneration of internal structures in the bulbous masses (alone), the amputation of all three masses resulted in blastema formation and appeared to regenerate missing bulbous mass tissue (Figure 2E; Table 2).

**TABLE 2:** Bulbus mass regeneration phenotypes.

Ectopic Pattern	Number amputated	% Forming blastema	% Regenerated	% Regeneration of Bulbus mass^a^	% Ectopic limb formation
Bulbus mass	3	100	100	100%	0
Bulbus mass with ectopic limb	6	100	100	n/a	100

^a^ Regeneration of bulbus mass implies the generation of tissue beyond the plane of amputation.

While these results show that both types of bulbous masses (those with and without ectopic limbs) retain the ability to regenerate, there are clear differences in their abilities to form ectopic limb structures, and thus, the composition of positional information in these tissues is likely different. The regenerative assay described above shows that bulbous masses that are connected to ectopic limbs have limb positional information, while it remained unclear whether bulbous masses without limb structures had this information or not. The failure of bulbus masses (alone) to generate limb structures could be explained by the lack of positional diversity (either only anterior or posterior limb information) as in Nacu et al. (2016), or that they contain only flank positional information as in Hirata et al. (2013), or have relatively less innervation than the bulbus masses connected to ectopic limbs.

**FIGURE 3:**
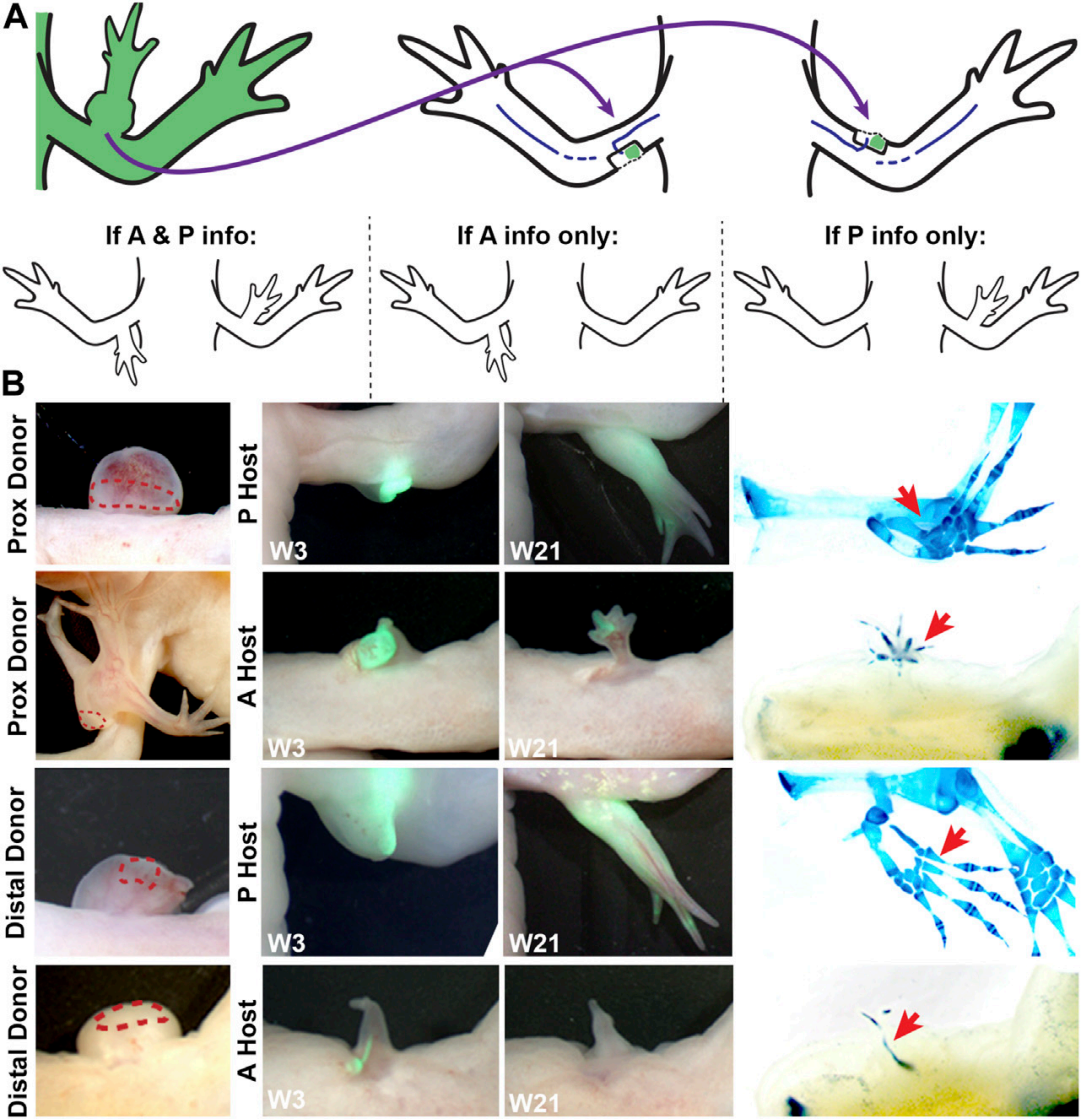
Characterizing anterior/posterior positional information in the bulbous mass using the ALM assay. **(A)** Cartoon representing the surgical procedure where bulbous mass tissue form a GFP + animal (green) is grafted to innervated anterior or posterior limb wound sites. Cartoons below explain the possible phenotypes in this assay and how they are interpreted. **(B)** Live images of the bulbous masses where grafts were obtained form (red dotted line outlines the location where the graft was taken) at 3 and 21 weeks (W3, W21) post-surgery. Host locations are indicated; where “P host” is a posterior wound site, and “A host” is an anterior wound site. (Right) Images of whole mount skeletal preparations of ectopic limb structures (red arrows). Full phenotype data is provided in Table 3.

To test the above idea, and to better characterize the limb positional information in the bulbous masses with ectopic limbs, we performed a series of grafting experiments using the accessory limb model (ALM) assay (Figure 3; Table 3). The ALM assay consists of grafting tissue from one side of the A/P limb axis into an innervated wound site on the opposing side of the limb, which will induce the formation of ectopic limbs if the grafted cells have opposing positional information with the host site (Endo et al., 2004; McCusker et al., 2016; Nacu et al., 2016). This inductive ability can be leveraged to assay whether a tissue has anterior and/or posterior positional information; tissue grafts with anterior information will induce ectopic limbs in posterior located sites, and vice versa (reviewed in (Raymond and McCusker, 2022)) (Figure 3A).

**TABLE 3:** Ectopic pattern induction by bulbus mass tissue.

Donor graft	Host location	ALMs Performed	ALMs Counted^a^	No induction	Single nodule	Rod	Multiple asymmetrical	Complete limb
Proximal	Posterior	30	20	5 (25%)	1 (5%)	1 (5%)	1 (5%)	12 (60%)
	Anterior	15	13	10 (77%)	2 (15%)	0	1 (8%)	0
Distal	Posterior	35	25	11 (44%)	3 (12%)	0	2 (8%)	9 (36%)
	Anterior	16	12	9 (75%)	2 (17%)	0	1 (8%)	0

^a^ ALMs, that lost the host graft or did not form a blastema within 3 weeks were excluded from further analysis.

Thus, bulbous mass tissue from GFP + axolotl was grafted into both posterior and anterior innervated wound sites on white host animals and the induction of limb structures were assessed visually through weekly documentation and by whole mount skeletal staining 21 weeks after the grafting surgery (Figure 3B). To account for possible differences in the distribution of positional information in the bulbous mass (ex. there might be more positional diversity in the region closest to the ectopic limb), we grafted tissue from either the distal or proximal region of the bulbous masses (Figure 2D). We observed that the grafts were only able to induce complete limb structures when grafted into posterior-located wound sites (Figure 3B; Table 3). Additionally, as opposed to the donor tissues, the ectopic structures that formed did not have bulbus masses, and were well connected to the host tissue. In contrast, the grafts induced simple structures in anterior located wounds. We did not observe differences in inductive abilities in the grafts obtained from either the proximal or distal regions of the bulbous mass. However, the bulbus masses connected to ectopic limbs had increased inductive activity than those without (60% compared to 36%, respectively, forming complete limbs).

These observations indicate that differentiated bulbous mass tissue is largely composed of cells with anterior-limb positional information. The regeneration assay in Figure 2 also supports this idea, because although amputations through the bulbus mass regenerate, the regenerated limb structure loses complexity, indicating a loss of positional diversity. This phenotype is different from our previous observations on the RA-induced ectopic limb tissue (distal to the bulbus mass), which indicate the presence of tissue with positional information from both sides of the A/P axis (Vieira et al., 2019). The asymmetrical distribution of A/P information along the P/D axis of the ectopic tissue is consistent with observations made by Gardiner and Bryant on the limb skeletal tissues. By documenting the induction of supernumerary limbs from limb amputations where the skeletal elements were rotated, they found that the stylopod element is composed of anterior, while the zeugopod contained both anterior and posterior information (Gardiner and Bryant, 1989).

### 3.3 Transcriptional characterization of bulbous mass tissue

**FIGURE 4:**
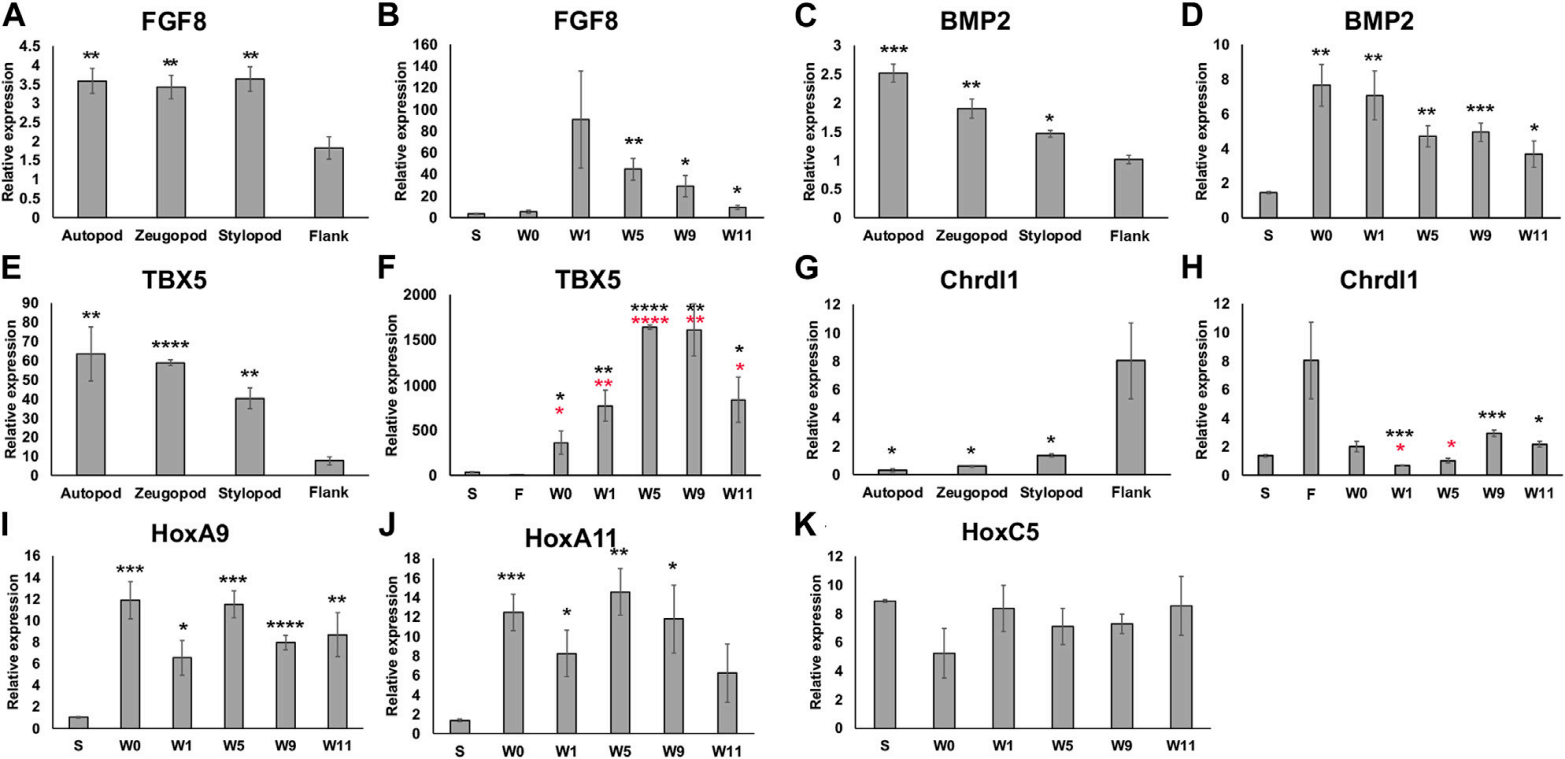
Expression of limb pattern genes in mature limb tissue and developing ectopic limb structures. **(A, C, E, G)** Histograms representing qRTPCR data for each primer set on mature limb tissue samples from the autopod, zeugopod, stylopod, and flank tissue. Statistical comparisons were made between the stump with each limb position using a two-tailed T-test with equal variance (*p < 0.05, **p < 0.005, ***p < 0.0005, ****p < 0.00005). **(B, D, F, H, I, J, K)** Histograms representing qRTPCR data for each primer set on limb tissue samples from stylopod (S), 7-day old innervated wound site zero, one, five, nine, and 11 weeks post RA injection (W0 –W11), and flank tissue **(F)**. Statistical comparisons between each time point and the stylopod (S) tissue are marked with black asterisk. In F and H comparisons between bulbous mass and flank tissue are marked with red asterisks. All data represents the average of 4 biological replicates. Error bars are SEM.

To further characterize the positional information in the anterior-located bulbous mass as it develops (at zero, one, five, nine, and 11 weeks post RA treatment) we performed an expressional analysis on blastema (*FGF8 and BMP2*) and positional markers (*Tbx5, Chrdl1, HoxA9, HoxA11, HoxC5*) using qRTPCR (Figure 4). 11 weeks post RA treatment was chosen as our final time point because at this stage, both the bulbous mass and ectopic limb structures had completed patterning and tissue differentiation (McCusker et al., 2014). Additionally, because the bulbous masses were located on the mid-stylopod of the forelimbs, we compared their expression with mature skin tissue samples from this region. We also harvested full-thickness skin (dermis and epidermis) from the autopod, zeugopod, and stylopod segments of the forelimb and flank tissue, to determine whether we could detect differences in expression of positional markers in mature tissue along the proximal distal limb axis.

We first analyzed the expression of both *FGF8* and *BMP2* as “blastema markers” because they are typically expressed in large abundances in the developing blastema (Guimond et al., 2010; Nacu et al., 2016), and we wanted to evaluate whether these markers remained elevated in the differentiated bulbous mass tissue at the latest time point collected (11 weeks). In both cases, gene expression was more abundant in the mature limb tissue compared to the flank (Figures 4A, C). When we compared the developing bulbous mass tissue with the mature stylopod tissue we found that expression of both genes was elevated during the early to mid-time points and remained elevated (although decreasing) to mature tissue-levels by week 11 (Figures 4B, D). Thus, the 11-week time point likely corresponds to the post-development stages of regenerative limb growth as described in Wells *et al* (Wells et al., 2021).

We next analyzed the expression of positional genes. We first evaluated the expression of *TBX5* and *Chrdl1* as general indicators of forelimb versus flank positional identities. We detected a graded expression of *TBX5* along the P/D limb axis, being most abundant in the autopod, and expression of all the limb samples were significantly greater than the flank tissue (Figure 4E). *Chrdl1*, showed the reverse expression pattern in the mature tissues, being significantly greater in the flank than all the limb samples (Figure 4G). *TBX5* was significantly elevated in all bulbous mass samples when compared to both mature stylopod and flank tissues (Figure 4F). Interestingly, at the later (differentiated) time points *Chrdl1* was expressed significantly more in the bulbous mass tissue compared to the mature stylopod but was not significantly different when compared to expression in the mature flank (Figure 4H). This data indicates that the bulbous mass is expressing markers of both forelimb (stylopod) and flank tissues.

Last, because the bulbous mass tissue expressed a forelimb marker (*TBX5*) and can generate limb tissues (Figures 2, 3), we were interested in comparing the expression of limb *Hox* genes between the bulbous mass tissue and the mature stylopod tissue. *HoxA9* was significantly elevated in the bulbous mass tissue compared to mature stylopod at all time points, including the last time point when the ectopic tissue was differentiated (Figure 4I). *HoxA11* expression was also significantly greater than stylopod in all the bulbus mass time points except for the Week 11 samples (Figure 4J). We note that de-integration of the bulbous mass tissue is typically noticeable by 3 weeks post RA treatment (Figure 2). Thus, our data shows disparities in *Hox* gene expression between the bulbous mass and the nearby stylopod tissue on the host limb during the times when de-integration is occurring. Interestingly, previous cross-grafting studies on mouse tibia and mandible, which express *HoxA11* or no *Hoxs* respectively, have shown that incongruencies in *Hox* expression also correlate with integration failure in this context (Leucht et al., 2008). We additionally analyzed the expression of *HoxC5,* a hox gene that is expressed in the flank of the developing vertebrate embryo in the region close to where the embryonic forelimb buds form (Nishimoto et al., 2014). No significant differences in *HoxC5* were detected between the sample groups (Figure 4K).

Apart from *HoxC5* and *Chrdl1*, none of the genes that we analyzed had significant differences in expression between the week 0 (pre-RA treatment) and week 1 (1 week post RA) samples. It was surprising that we did not detect a decrease in *FGF8* expression since a significant loss was previously detected by microarray in amputation blastemas treated with a retinoic acid receptor (RAR) agonist (Polvadore and Maden, 2021), and a *bona fide* repressive retinoic acid response element (RARE) has been identified upstream of the *FGF8* promoter in multiple vertebrate species (Kumar and Duester, 2014). We also identified multiple predicted RARE consensus sequences, including one that appears to align spatially with conserved inhibitory *FGF8* RARE in vertebrates, as well as in all the other axolotl genes that we analyzed (Supplementary Table S1). Although it is not yet known whether RARs bind to any of these predicted binding motifs to regulate gene expression, the presence of these sequences opens the possibility.

One possible explanation of why we did not detect significant changes in FGF8 expression following RA treatment is because our samples were collected 1 week following RA treatment, which could be sufficient time for the blastema to no longer be under the direct influence of the treatment. Additionally, our analysis was performed on anterior-located blastemas, as opposed to amputation blastemas, which were previously shown to express high abundances of *FGF8* (Nacu et al., 2016). Thus, it is also possible that part of the RA-dependent regulatory machinery that normally represses *FGF8* expression is not present in anterior located blastemas.

### 3.4 Positional information characterization of wound sites at the limb base

**FIGURE 5:**
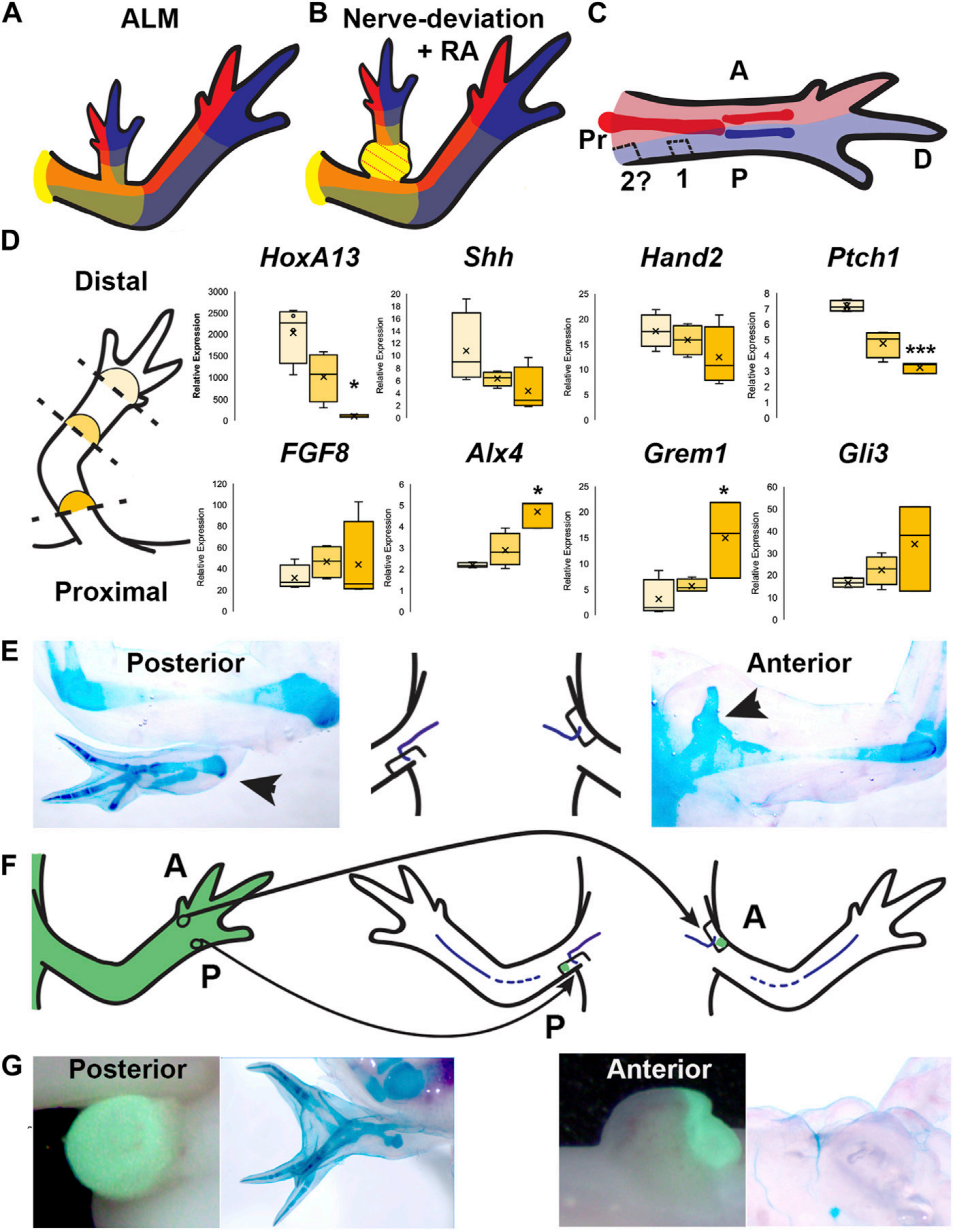
Distribution of positional information in RA-induced ectopic limbs and normal limbs. **(A, B)** Cartoon representing the hypothesized distribution of anterior (red), posterior (blue), and the P/D (gradient of yellow, strongest in proximal) positional memory in ALM ectopic limbs **(A)** and RA-induced ectopic limbs with bulbus mass **(B)**, and host limb tissues. Red dotted lines in bulbus mass indicate anterior positional information. **(C)** Cartoon representing the hypothesized distribution of A/P positional memories in the (internal) periskeletal tissue (dark red = anterior, dark blue = posterior) and (superficial) dermal tissue (light red and blue, respectively) in the uninjured limb. Squares indicate the hypothesized positional content in posterior ALM wound sites in mid-stylopod (1) and proximal stylopod (2) positions. **(D)** (left) Cartoon illustrating the localization of the blastemas collected for qRTPCR analysis. (right) Box and whisker plots of expressional data for *HoxA13, Shh, Hand2, Ptch1, FGF8, Alx4, Grem1, and Gli3* from autopod (light yellow), stylopod (mid-tone yellow) and zeugopod (dark yellow) blastema samples. Statistical comparisons between the autopod and zeugopod or stylopod were performed using a two-tailed T-test with equal variance (*p < 0.05, **p < 0.005). All data represents the average of 4 biological replicates. **(E)** Cartoon illustrating the positioning of the nerve deviated wound sites on posterior and anterior limb base locations, flanked by images of whole mount skeletal preparations of the most extreme growth phenotypes in each position**. (F)** Cartoon illustrating the engraftment of distal anterior **(A)** and posterior (P) full-thickness skin into nerve-deviated wound sites at the limb base. **(G)** (Left) Live images of nerve deviated posterior and anterior wound sites at the limb base 3 weeks post-surgery where GFP + distal tissue graft is green. (Right) Whole mount (WM) skeletal preparation of wound sites 15 weeks post-surgery. Phenotype statistics of all surgical manipulations are provided in Table 4.

Phenomenologically, the bulbous mass behaves like anterior limb tissue in the context of the ALM assay (Figure 3). In contrast, the same assay shows that the more distal RA-induced ectopic limb structures have both anterior and posterior positional information (Vieira et al., 2019), indicating that A/P positional information is asymmetrically distributed along the P/D limb axis (Figure 5). This trend is similar to that observed by Gardiner and Bryant in the limb skeletal tissues who discovered using pattern-induction assays that the humerus skeletal element has anterior information, while the more distal skeletal elements, the radius and ulna, have anterior and posterior information, respectively (Gardiner and Bryant, 1989). Interestingly, full-thickness skin from the posterior side of the mid-stylopod segment has been shown to contain posterior positional information in the ALM assay (Reviewed in (Raymond and McCusker, 2022) Combined, these observations indicate that the distribution of A/P information in the stylopod is also asymmetrically distributed along the A/P axis (Figures 5A–C).

We surmised that this asymmetry in A/P information in the limb axis could also translate to differential expression of A/P patterning genes in blastemas located in different positional along the P/D axis. To test this possibility, we performed qRT-PCR for A/P genes on mid-bud blastema samples collected from autopod, zeugopod, and stylopod-level amputations (Figure 5D). We first quantified the expression of *HoxA1*3, a distal limb marker (Gardiner et al., 1995), and detected significantly less *HoxA13* in the most proximal compared to most distal-located blastemas as expected. We next evaluated the expression of *Shh*, *Hand2,* and *Ptch1* (posterior) and *FGF8, Alx4, Grem1,* and *Gli3* (anterior) (Figure 5D) (Nacu et al., 2016; Vieira et al., 2019). For all three “posterior” genes we detected a similar P/D expression trend, where *Ptch1* showed significantly more expression in the distal blastemas. All four anterior patterning genes exhibited the opposite trend, with more abundant expression in the proximal blastemas, and this trend was significant with *Alx4* and *Grem1* (Figure 5D)*.* These data are consistent with the idea that the asymmetrical distribution of A/P information along the P/D limb axis corresponds with subsequent differences in A/P gene expression in blastemas that form in these locations. It is possible that these differences in gene expression play a role in generating the A/P pattern that is appropriate to position of the limb amputation.

The observations that the bulbus mass has an anterior limb identity (Figure 3; Figure 5B) and that both mature and blastema tissue from the stylopod have relatively more anterior information than in the distal limb regions (Gardiner and Bryant, 1989; Vieira et al., 2019). (Figure 5), led us to hypothesize that the anterior boundary may approach the posterior side of the limb at the limb base Figure 5C). To test the above possibility, we again leveraged the ALM assay to evaluate the natural distribution of A/P positional information in most proximal-limb regions (Figures 5E–G; Table 4).

**TABLE 4:** Ectopic pattern induction in nerve deviated limb base wounds.

Surgery location/type	Number performed	Surgeries scored	Formed epiphysis bulge	Formed ectopic limb structures
Posterior limb base	10	7^a^	0	1 (14%)
Posterior limb base with distal graft	10	7^b^	0	3 (43%)
Anterior limb base	8	8	4 (50%)	0
Anterior limb base with distal graft	9	9	3 (33%)	0

^a^ 3 of the surgeries did not develop a blastema and were excluded from further analysis.

^b^ 3 of the surgeries did not develop blastemas and/or tissue grafts were undetectable by 3 weeks post-surgery and were excluded from further analysis.

Previous studies showed that deviating a nerve to a wound site on the mid-stylopod, in either anterior or posterior locations, fails to induce ectopic limb structures due to the lack of positional information from the opposite side of the A/P axis (Endo et al., 2004; Phan et al., 2015; Nacu et al., 2016). However, if the anterior boundary approaches the posterior side of the limb the limb base, we expected nerve deviated wounds in this location to have increased potential to generate ectopic limb structures compared to the anterior side of the limb (Figures 5C,E–G). We deviated nerves to anterior and posterior wound sites at the limb base and allowed to develop for 11 weeks before harvesting for whole mount skeletal staining. 0% of the anterior located blastemas formed ectopic limb structures, however we did observe small cartilage bulges fused to and extending from half of the proximal humerus epiphyses (Figure 5E; Table 4). We suspect that, because of their location, these cartilage bulges are ectopic outgrowths from the growth plate, potentially stimulated by the mitogens generated in the blastema. In contrast 14% (1 of 7) of the posterior limb base blastemas generated ectopic skeletal elements with complex structure and appeared to contain both zeugopod elements (Figure 5E; Table 4). We note that a similar phenotype was previously reported by Bodemer in nerve deviated wounds at the limb base (Bodemer, 1958).

We surmised two feasible explanations for the low penetrance of ectopic limb induction in the posterior sites, 1) that the outcome was due to technical variations in the surgery, or 2) that the posterior limb base may predominantly have either anterior or posterior positional information. To further test this idea, we grafted anterior or posterior skin tissue from the autopod into anterior or posterior limb-base wound sites, respectively. Because the A/P boundary is more centralized in the distal limb regions (Gardiner and Bryant, 1989; Vieira et al., 2019) (Figure 5C), we predicted that the distal posterior skin grafts would induce ectopic limbs in the posterior limb base sites if the site contained “anterior” positional information. We observed that 43% of the grafted posterior wounds, and 0% of the grafted anterior wounds, formed complex (multiple element) structures (Figures 5F,G; Table 4). We interpret this data to indicate that skin wounds in the posterior region of the limb base contain anterior positional information. However, the grafted wounds appear to have a lower degree of positional diversity because the ectopic limb structures that form appear to be symmetrical and are missing more proximal elements. Studies from the Satoh lab have shown that grafts of distal skin in the ALM induce the generation of ectopic autopod structures (Makanae et al., 2014b).

Together these observations provide a more complete map of the A/P boundary in the limb and could contribute to our understanding of the specification of positional information during limb development in embryonic and adult axolotl.

## 4 Conclusion

The primary objective of this study was to better understand the underlying cause of integration failure between RA-induced ectopic structures, specifically with the bulbous mass, and the host limb. We hypothesized that either irreconcilable differences in positional information or mechanical (shear) forces generated by the bulbous mass itself could lead to the diminishment of integration with the host limb. After ruling out the possible contribution of mechanical forces, we focused on characterizing the positional information in the bulbus mass and the host sites to determine whether differences in this information could account for integration failure. By performing regenerative assays (amputation and grafting into ALMs) we determined that the bulbous mass contains anterior limb positional information. Thus, incongruencies in A/P information are unlikely to drive integration failure of the bulbus mass because the bulbus mass and the host site both contain anterior positional identities (Figure 5B). Expressional analyses for positional markers exhibited significant differences in expression in the bulbus mass relative to the region of the host limb in which it was located. This data indicates that mismatched positional information coincides with integration failure in the bulbus mass, however further work will be needed to determine whether the positional “identity” of the bulbus mass corresponds to a specific location on the axolotl body or not. In the future, it will be informative to evaluate the above-described properties and how they correlation with tissue integration using decreasing doses of RA since it has been well-established that RA has a dose-dependent effect on the proximalization of limb pattern (Thoms and Stocum, 1984). Combined, these studies establish the bulbous mass tissue as a unique system to further study integration failure of both soft and skeletal/joint limb tissues.

The unexpected observation that the bulbus mass tissue contained predominantly anterior positional information, combined with previously published observations, led us to the hypothesis that the A/P boundary extends to the posterior side of the limb in the limb base. Expressional analyses show that proximal blastemas express more anterior, and less posterior, patterning genes than distally located blastemas. Using the ALM assay, we found that grafts of distal posterior skin into posterior wounds at the limb base results in the induction of ectopic limb tissue, which does not happen in anterior wounds. These findings provide new insight into the P/D distribution of A/P positional information, which could have important implications in our understanding of how the location-appropriate limb pattern is regenerated.

## Data Availability

The raw data supporting the conclusion of this article will be made available by the authors, without undue reservation.
